# Complex Perineal Trauma with Anorectal Avulsion

**DOI:** 10.1155/2016/4830712

**Published:** 2016-11-07

**Authors:** Adelina Maria Cruceru, Ionut Negoi, Sorin Paun, Sorin Hostiuc, Ruxandra Irina Negoi, Mircea Beuran

**Affiliations:** ^1^Emergency Hospital of Bucharest, Bucharest, Romania; ^2^Carol Davila University of Medicine and Pharmacy, Bucharest, Romania; ^3^National Institute of Legal Medicine Mina Minovici, Bucharest, Romania

## Abstract

*Introduction*. The objective of this case report is to illustrate a severe perineal impalement injury, associated with anorectal avulsion and hemorrhagic shock.* Results*. A 32-year-old male patient was referred to our hospital for an impalement perineal trauma, associated with complex pelvic fracture and massive perineal soft tissue destruction and anorectal avulsion. On arrival, the systolic blood pressure was 85 mm Hg and the hemoglobin was 7.1 g/dL. The patient was transported to the operating room, and perineal lavage, hemostasis, and repacking were performed. After 12 hours in the Intensive Care Unit, the abdominal ultrasonography revealed free peritoneal fluid. We decided emergency laparotomy, and massive hemoperitoneum due to intraperitoneal rupture of pelvic hematoma was confirmed. Pelvic packing controlled the ongoing diffuse bleeding. After 48 hours, the relaparotomy with packs removal and loop sigmoid colostomy was performed. The postoperative course was progressive favorable, with discharge after 70 days and colostomy closure after four months, with no long-term complications.* Conclusions*. Severe perineal injuries are associated with significant morbidity and mortality. Their management in high volume centers, with experience in colorectal and trauma surgery, allocating significant human and material resources, decreases the early mortality and long-term complications, offering the best quality of life for patients.

## 1. Introduction

Severe rectal injuries are rarely met into the clinical practice even in tertiary emergency centers, but when present, they are usually associated with significant morbidity and mortality. Secondary to the abundance of blood vessels and nervous structures into the pelvis, patients commonly present severe hemorrhage and nervous injuries. Surgical approach of patients with rectal injuries is an ongoing subject of debate, while the classical recommendations of proximal fecal diversion, irrigation, and large drainage of the presacral space are based on data collected in World War II and Vietnam War [1]. When facing severe pelvic and perineal injuries, the trauma surgeon should choose appropriate management according to the degree of rectal wall destruction, fecal contamination, hemodynamic status, and pelvic instability [2, 3].

The objective of this case report is to illustrate a severe perineal penetrating injury, associated with massive soft tissues pelvic destructions, hemorrhagic shock, and long-term morbidities.

## 2. Case Report

**Figure 1:**
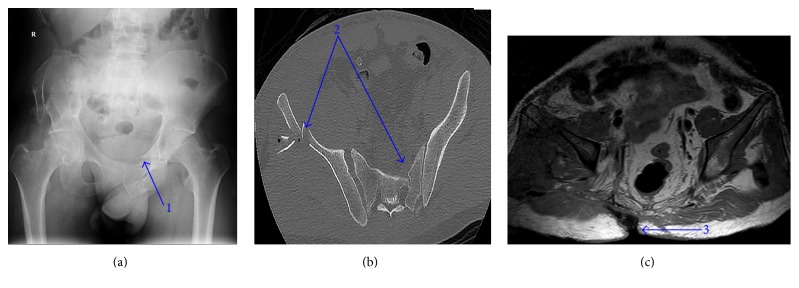
(a) X-ray image of the pelvic fracture four months after trauma (1); (b) Computed Tomography in the fifth day from admission revealing the fracture of the pelvis (2); (c) magnetic resonance imaging 40 days from admission demonstrating the remnant of the perineal wound defect (3).

**Figure 2:**
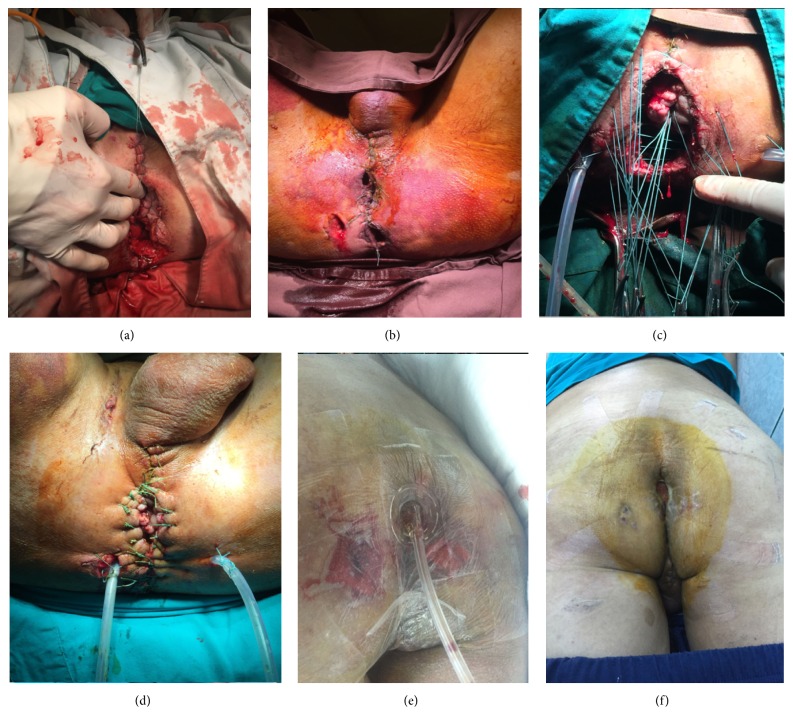
Illustration of the perineal wound. (a) On first surgical procedure in our center; (b) 48 hours later before packs removal; (c) and (d) 48 hours later, after packs removal, lavage, reinsertion of the anorectum, and large drainage of the pelvic space; (e) negative wound pressure therapy of the perineal wound; (f) healed perineal wound on discharge.

A 32-year-old male fireman, was referred to our hospital from a regional county hospital, for an impalement work-related perineal and pelvic trauma, associated with complex pelvic fracture and massive perineal soft tissue destruction and anorectal injury. In the referring hospital, the patient was transported directly to the operating room due to severe hemorrhage and perineal packing performed. On arrival, the patient had a systolic blood pressure of 85 mm Hg and a hemoglobin value of 7.1 g/dL (Figure 1). The patient was transported to the operating room, and perineal lavage, hemostasis, and repacking were performed (Figure 2).

After 12 hours in the Intensive Care Unit, the abdominal ultrasonography revealed free peritoneal fluid of 47 mm diffuse distributed throughout all abdominal quadrants, which was confirmed to be blood on diagnostic peritoneal lavage. To exclude intra-abdominal bleeding, we decided emergency laparotomy. We observed a massive hemoperitoneum due to intraperitoneal rupture of pelvic hematoma. Pelvic packing controlled the ongoing diffuse bleeding. After 48 hours, the relaparotomy with packs removal and loop sigmoid colostomy was performed, to prevent contamination of the perineal wound. Pelvic Computed Tomography in the fifth day of admission revealed multiple pelvic fractures of the left sacrum wing, right iliac wing, bilateral superior pubis rami and ischiopubic rami, and right sacroiliac dislocation.

The microbiological exam of the perineal wound secretions on the tenth postoperative day revealed* Escherichia coli* and on the 34th postoperative day showed* Proteus* spp.,* Klebsiella* spp., and* Pseudomonas* spp. The patient received as antibiotic therapy Imipenem/Cilastatin, 2 grams per day for 22 days, Linezolid, 1200 mg per day for 14 days, Colistin, 6,000,000 IU per day for ten days, and Metronidazole, 2 g per day for 15 days. The postoperative course was progressive favorable, with a daily dressing of the perineal wound during the first 14 days and negative wound pressure therapy after that.

The patient was discharged after 70 days. At four months, the clinical exam confirmed the functionality of the anal sphincter muscle and the colostomy closed. There were no long-term complications. The patient's follow-up at six months revealed no impairment of the anal continence.

## 3. Discussions

We presented a case of an active young man with severe perineal penetrating trauma and anorectal injury successfully managed by a multidisciplinary team highly experienced in colorectal and trauma surgery.

The anorectal avulsion represents a rare and highly morbid injury, usually associated with severe pelvic trauma. A systematic review of the English-language literature revealed only few case reports about the theme (Table 1).

Fecal diversion, far from being a surgical dogma, represents one of the most important surgical maneuvers to address an extraperitoneal rectal injury, especially in destructive perineal lesions [11–14]. Ulger et al. evaluated the benefits of ostomy in 63 patients with rectal injuries, managed between 2000 and 2011 [15]. The authors concluded that primary repair is safe in selected patients with grade II intra- or extraperitoneal rectal injuries, while colostomy should be appropriate for patients with sphincter injury, fecal contamination, or long trauma treatment interval [15]. From 3442 patients treated during Operation Iraqi Freedom, 175 had colorectal lesions [16]. Stomas were used more frequently with rectal or sphincter injuries (25/40–65%). Rectal injuries (odds ratio = 22, *p* = 0.03) were independently associated with increased mortality on multivariate analysis [16]. Watson et al. reviewed the US Department of Defense Trauma Registry, which included 867 military personnel with colorectal injuries [17]. The higher diversion rate was found for rectal injuries (56%). On multivariate analysis, significant predictors for stoma creation were rectal involvement (odds ratio (OR) = 2.2, rectum versus left colon; OR = 7.5, rectum versus right colon), gunshot wounds (OR = 3.4), Injury Severity Score ≥ 16 (OR, 1.7), and damage control surgery (OR, 1.6) [17]. The results of Glasgow et al., which present 977 coalition military personnel registered during eight years with colorectal injuries, should be noted [18]. The authors found that mortality was significantly higher for rectal injuries managed without fecal diversion (10.8% versus 3.7%, *p* < 0.0001) [18].

Although the evidence regarding the benefits of presacral drainage is lacking, we are usually widely draining the blunt and impalement injuries of the rectum. The only randomized controlled trial, addressing the penetrating rectal injuries, concluded that presacral drainage had no effect on infectious complications [19]. The same results come from Brown et al., who studied 57 patients with penetrating rectal injury sustained in Iraq and Afghanistan [20]. Complications were found in 21% of patients, but logistical regression failed to show a correlation between these complications and presacral drainage (*p* = 0.9) and distal washout (*p* = 0.33) [20]. We think that in anorectal avulsions with severe perineal soft tissue destructions, presacral drainage should be used, having significant clinical benefits.

The angiography is a very useful adjunctive measure to stop the bleeding in patients with severe pelvic trauma [21]. We preferred not to use it in the presented case, considering the severe soft tissue destruction as the primary source of the hemorrhage. On the other hand, the angiography with embolization only controls the arterial bleeding, being useful in 3%–10% of patients with pelvic fracture [21].

Russell et al. evaluated the long-term fecal continence in children with injuries requiring surgical repair of the anal sphincters [22]. Out of 21 patients, 90% were continent at the last follow-up [22]. Our patient also has no impairment of the fecal continence, despite severe destructions of the pelvic and soft perineal tissues.

## 4. Conclusions

Severe perineal injuries are associated with significant morbidity and mortality. Their management in high volume centers, with experience in colorectal and trauma surgery, allocating significant human and material resources, decreases the early mortality and long-term complications, offering the best quality of life for patients.

## Figures and Tables

**Table 1:** Systematic review of the English language literature regarding anorectal avulsions.

Reference	Trauma kinetics	Injuries pattern	Emergency surgery	Quality of life
Mathieson and Mann, 1965 [4]	Farmworker fell in front of a caterpillar tractor which passed across his body	Bilateral fractures of the superior pubis rami and ischiopubic rami, complete rupture of the posterior urethra, anorectal avulsion	Realignment of the urethra, cystostomy, loop sigmoid colostomy, reinsertion of the anorectum, drainage of the pelvis space	At one year, complete continence for stool and flatus
Sharma et al., 2000 [5]	Riding a bicycle and being hit by a high-speed truck from behind	Anorectal avulsion, fracture of both inferior rami of the pubis	Sigmoid loop colostomy, suprapubic cystostomy, anatomical repair of the perineum, presacral drainage	Colostomy closed after four months; discharged after seven months, normal continence
Terrosu et al., 2011 [6]	Lying on his back and heavy scaffolding fell on him	Anal avulsion, deep laceration of the left lumbar area, urethral rupture, severe pelvic fracture	Suprapubic cystostomy, levator ani reconstruction, packing of the lumbar wound, fixation of the pelvis, reimplantation of the anus, pelvic drainage, transverse colostomy	24 months after the accident, complete continence, normal urological and sexual function, residual motor and sensory deficit in his left lower extremity
Rispoli et al., 2012 [7]	Motorcycle crash, probably impalement	Splenic injury, vertebral fractures, multiple rib fractures, fracture of the left inferior rami of the pubis, longitudinal fracture of the sacrum and coccyx	Splenectomy, sigmoid loop colostomy, presacral drainage. After 24 hours, reattachment of the anorectum was impossible, conservative management	At three years, no incontinence, a complete return to normal life, anal canal with normal tone but dislocated cranially
Ibn Majdoub Hassani et al., 2013 [8]	Not specified (accident)	Pelvic fracture, rib and spine fractures, anorectal avulsion	Suprapubic cystostomy, rectal washout, necrosectomy, presacral irrigation, primary repair of the perineum, presacral drainage, sigmoid loop colostomy	At six months, no physiologic dysfunction on anorectal manometry; anal stenosis requiring dilatations
Gomes et al., 2013 [9]	Motorcycle accident, partially run over by a vehicle over the right side of the pelvis	Right superior and inferior pubic rami fracture, T11 transverse process fracture. On survey in 72 hours, anorectal avulsion	Diverting sigmoid loop colostomy; reimplantation of the anorectum was not possible.	On four weeks, no tone of the anal sphincter, then loss to follow-up
Page et al., 2015 [10]	Motor vehicle collision with ejection	Complete anorectal dissociation, pelvic floor destruction	Diverting colostomy, perineal washout; hospital day five, completion proctectomy and rectus abdominis myocutaneous flap	Permanent stoma
